# The Role of Percutaneous Transjugular Portosystemic Shunts

**DOI:** 10.1155/1992/64526

**Published:** 1992

**Authors:** Roy L. Gordon

**Affiliations:** University of California 505 Parnassus Avenue San Francisco California 94143-0628 USA


### THE ROLE OF PERCUTANEOUS TRANSJUGULAR PORTOSYSTEMIC SHUNTS

### ABSTRACT

*Zemel, G., Katzen, B.T., Becker, G.J., Benenati, J.F. and Sallee, D.S. (1991) Percutaneous transjugular portosystemic shunt. Journal of American Medical Association* **266**: 390–393.

**Objective. — To determine the effectiveness of the Palmaz balloon expandable stent for the creation of a transjugular intrahepatic portosystemic shunt. The device is designed to achieve portal decompression in patients with variceal hemorrhage secondary to portal hypertension.**

**Design. — Transjugular intrahepatic portosystemic shunting was performed in eight patients during a 9-month period. Mean follow-up was 5 months.**

**Patients. — All patients had cirrhosis with portal hypertension and varices. Bleeding occurred in seven patients from esophageal varices and in one patient from hemorrhoids.**

**Main Outcome Measures. — Shunt patency and recurrent variceal hemorrhage.**

**Results. — Shunts created from a transjugular approach between a hepatic and a portal vein (diameters of 8 to 12 mm) lowered the average portosystemic pressure gradient from 36 to 11 mm Hg. Mean postoperative hospital stay was 7.7 days. Complete variceal decompression after transjugular intrahepatic portosystemic shunt placement was identified endoscopically in all eight patients. The patient treated for hemorrhoids rebled and was treated successfully by transfemoral balloon expansion of the shunt diameter from 8 to 12 mm. All shunts were patent at 1 to 9 months (mean, 5 months) of follow-up.**

**Conclusion. — Initial results suggest that transjugular intrahepatic portosystemic shunt is a safe and effective method of portal decompression for the treatment of variceal hemorrhage.**

### PAPER DISCUSSION

KEY WORDS: Portosystemic shunt, oesophageal varices

The creation of a transjugular intrahepatic portosystemic shunt (TIPS) in experimental animals was first described by Rosch, Hanafee and Snow in 1969[1]. Although they could establish these shunts, they could not solve the problem of keeping the shunt patent. In 1982, Colapinto *et al.* described using the technique in humans utilizing balloon dilatation of the intrahepatic tract, but they too were unsuccessful in achieving durable patency of the shunt[2]. The introduction of the metallic intravascular stent provided the technological advance needed to maintain patency of the intrahepatic shunt, described in animals by Palmaz *et al.* in 1985[3] and in humans by Richter *et al.* in 1990[4].

Using a metallic stent, TIPS offers the promise of providing a simple, effective non-operative method for treating portal hypertension by means of intrahepatic portosystemic shunting. The first patients were treated by Richter in Germany using the Palmaz stent[4]. Excellent results were achieved but the early technique was complex and lengthy, involving both a transjugular and a transhepatic approach. This group now has treated over 50 patients.

The paper by Zemel *et al.* from Miami is the first published series in the USA using the Palmaz stent. The excellent results achieved in their first eight patients corroborate the results achieved by Richter's group using the Palmaz stent.

Other vascular stents are available and at UCSF we have employed the more flexible Wallstent[R] in over 50 patients[5]. The Wallstent[R] is somewhat easier to employ and is perhaps more versatile than the Palmaz stent. The key question, however, will concern long-term patency of the shunt and whether one stent type is better than another in this respect.

Zemel *et al.* reported a 100% assisted primary patency rate. This involved second procedures in one patient 19 days after initial shunting and in two patients 4 months after initial shunting. The first patient rebled, but the authors were able to successfully dilate the original stent from a diameter of 8 mm to 12 mm. This reduced the pressure gradient and bleeding ceased. Since blood flow is proportional to the fourth power of the radius of the stent a diameter increase from 8 to 12 mm will produce an increase of about 500% in bloodflow through the shunt. Narrowings in the hepatic vein segment of the shunts of the other two patients were also successfully treated by percutaneous transvenous balloon dilatation.

It therefore appears that not only can TIPS be placed simply and safely by percutaneous techniques but, in addition, the shunt can be enlarged or modified at a later stage by equally simple percutaneous techniques. Further developments may perhaps even allow the shunt to be narrowed at a later stage if encephalopathy is a problem. A better understanding of the cause of post-shunting liver failure and encephalopathy may emerge if variable partial shunting becomes practical.

At this preliminary stage of clinical investigation the optimal shunt diameter has not yet been established. Should shunt diameter be selected on the basis of achieving a target pressure gradient across the shunt; should it be based on angiographic studies showing no flow to varices; should the aim be to maintain flow to the liver; or should a smaller shunt diameter be chosen initially to minimize potential encephalopathy reserving the option of subsequent stent dilatation to increase shunting if bleeding recurs? Current techniques employ standard vascular stents that are dilatated up to the required diameter of 8-, 10- or 12 mm using the appropriate sized balloon catheter. These sizes were used because these were the currently available sizes that were in use in the arterial tree and the biliary tract.

It may become clear that larger stents have to be developed or that stents placed in parallel using two hepatic veins produce better results. Work by Rypins and Sarfeh and by Johansen suggest that small diameter surgical shunts in the 8 to 12 mm range are effective at decompressing portal hypertension and have low rates of encephalopathy[6,7].

The indications for shunting in Zemel's study were variceal bleeding as a result of portal hypertension in seven of eight patients and hemorrhoidal bleeding in one patient. The severity of cirrhosis was classified as 2 Childs A, 5 Childs B, and 1 Childs A. In our initial 42 patients treated at UCSF the patients had much more severe cirrhosis, with 2 Childs A, 8 Childs B, and 34 Childs C. Two patients were successfully treated for intractable ascites, but the remainder all had recurrent variceal bleeding after sclerotherapy. Sixteen patients were actively bleeding at the time of shunting and 24 had suffered recurrent bleeds (average 3.6 episodes) within the previous few days. Fifteen of our 16 actively bleeding patients ceased bleeding at the time of shunt placement. The sixteenth patient continued to bleed and endoscopy revealed a bleeding duodenal ulcer. The shunt was patent and there was no variceal bleeding. This experience suggests that TIPS may be a good treatment for both acute and recurrent variceal bleeding and we are embarking on a prospective randomized clinical trial comparing TIPS and sclerotherapy.

Zemel *et al.* mention an additional role for TIPS in the treatment of patients with end-stage liver disease who are candidates for liver transplantation. The first 13 patients whom we treated at UCSF were in this category[8]. In all these patients TIPS provided acute control of variceal bleeding eliminating the need for emergency transplantation. Time was thus available to find suitable liver donors and the surgery of transplantation was not complicated by prior shunt surgery. Seven of these patients successfully underwent subsequent liver transplantation. The availability of the excised livers was a great windfall allowing histopathologic study of the shunt at various stages of maturity ranging from four days to three months[9]. It appears that by three weeks the luminal surface of the stent had become covered by a thin layer of granulation tissue with a single layer of endothelial cells. In a sense, an artificial blood vessel had been created within the liver parenchyma with the metallic stent as a scafold.

As shown by Zemel *et al.* the transjugular route appears safe and the shunts effective. The next few years promise great advances in this field not only in the treatment of portal hypertension and bleeding, but also in the histopathologic study of intrahepatic stents in transplant patients. Direct access to the portal system provides the potential for significant physiological studies in many fields of portal dynamics and liver function.

### REFERENCES

1. Rosch, J., Hanafee, W.N. and Snow, H. (1969) Transjugular portal venography and radiologic portocaval shunt: An experimental study. *Radiology*, **92**, 1112–1114
2. Colapinto, R.F., Stronell, R.D. and Birch, S.J. *et al.* (1982) Creation of an intrahepatic portosystemic shunt with a Gruntzig balloon catheter. *Can. Med. Assoc. J.*, **126**, 267–268
3. Palmaz, J.C., Sibbitt, R.R. and Reuter, S.W. *et al.* (1985) Expandable intrahepatic shunt stent: early experience in the dog. *A.J.R.*, **145**, 821–825
4. Richter, G.M., Noeldge, G. and Roessle, M. *et al.* (1990) Transjugular intrahepatic portosystemic stent shunt (TIPPS). *Radiology*, **174**, 1027–1030
5. LaBerge, J.M., Ring, E.J. and Gordon, R.L. *et al.* (1992) Transjugular intrahepatic portosystemic shunts (TIPS): Preliminary results in 25 patients. *J. Vasc. Surg.* (in Press)
6. Rypins, E.B. and Sarfeh, I.J. (1990) Small-diameter portacaval H-graft for variceal hemorrhage. *Surg. Clin. N. Amer.*, **70**, 395–404
7. Johansen, K. (1989) Partial portal decompressin for variceal hemorrhage. *Am. J. Surg.*, **157**, 479–482

8. Ring, E.J., Lake, J.R., Roberts, J.P. and Gordon, R.L. (1992) Intrahepatic portosystemic shunts to control variceal bleeding prior to liver transplantation. *Ann. Int. Med.* 1992; 116: (In Press)
9. LaBerge, J.M., Ferrell, L.D., Ring, E.J. and Gordon, R.L. *et al.* (1992) Histopathology of transjugular intrahepatic portosystemic shunts. *Journal of Vascular and Interventional Radiology* JVIR 1991; 2: 54–56

Roy L Gordon
Professor of Radiology
University of California
505 Parnassus Avenue
San Francisco
CALIFORNIA 94143–0628
United States of America

